# Uncommon Locations of Cystic Echinococcosis: A Report of 46 Cases from Southern Iran

**DOI:** 10.1155/2020/2061045

**Published:** 2020-09-18

**Authors:** Reza Shahriarirad, Amirhossein Erfani, Mehrdad Eskandarisani, Mohammad Rastegarian, Bahador Sarkari

**Affiliations:** ^1^Thoracic and Vascular Surgery Research Center, Shiraz University of Medical Sciences, Shiraz, Iran; ^2^Student Research Committee, Shiraz University of Medical Sciences, Shiraz, Iran; ^3^Basic Sciences in Infectious Diseases Research Center, Shiraz University of Medical Science, Shiraz, Iran; ^4^Department of Parasitology and Mycology, School of Medicine, Shiraz University of Medical Sciences, Shiraz, Iran

## Abstract

**Background:**

Most cases of hydatid cysts form in the liver and lung and other tissues are considered as unusual locations in hydatid cysts. The current study aimed to find out the rate and features of hydatid cysts in uncommon locations in Fars Province, Southern Iran, over a 15-year period.

**Methods:**

The hospital records of patients who underwent surgery for hydatid cysts in university-affiliated hospitals in Fars Province, Southern Iran, from 2004 to 2018, were retrospectively reviewed. For each patient, clinical and demographical data were recorded.

**Results:**

During a 15-year period, a total of 501 patients were surgically treated for hydatid cysts, and out of these, 46 (9.2%) were presented with the unusual locations of hydatid disease. Males constituted 28 (60.9%) of these patients while 18 (39.1%) of the patients were females. The patients' age ranged from 5 to 80 years (mean = 40.49; SD = 20.37). The size of the cysts ranged from 2 to 20 cm (mean = 8.69, SD = 4.59). The most common unusual location for the hydatid cyst was the spleen with 30.4% of cases, followed by the pelvic cavity (15.2%). Out of 46 cases with unusual location of the hydatid cyst, 10 (21.7%) cases had lung, 22 (47.8%) cases had liver, and 5 (10.9%) cases had both liver and lung hydatid cysts, simultaneously with cysts in unusual locations.

**Conclusion:**

In cystic echinococcosis- (CE) endemic areas, hydatid disease can affect any organ, from head to toe, in humans. The disease should be considered in the differential diagnosis of any cystic entities anywhere in the body.

## 1. Introduction

Cystic echinococcosis (CE) or hydatid cyst is a zoonotic parasitic disease caused by the larvae of *Echinococcus granulosus*. The disease is widespread worldwide and is very common in the Middle East countries, including Iran, Iraq, and Turkey [1–3]. Dogs and other canine act as the definitive hosts of the parasite, while sheep, cattle, goats, and camels are considered as its intermediate hosts. Humans may accidentally become infected through ingesting water or food contaminated with parasite eggs that have been excreted in the feces of dogs into the environment [4]. Once ingested, the larvae enter the bloodstream, from where they are drawn to various parts of the body, where they grow up and become the hydatid cysts. Although most cases of hydatid cysts form in the liver (60–70%) and the lungs (20%), all of the human body organs can be affected by this disease [5]. Tissues other than the liver and lungs are considered as unusual locations in hydatid cysts. Unusual locations of hydatid cysts include, but not limited to, the spleen, peritoneal and pelvic cavities, central nervous system (CNS), bone, pancreas, eye, kidney, heart, ovary, and salivary glands [5–8]. Out of all unusual location sites of CE, osseous and orbital cases are very rare, accounting for only 0.5–2.5% and less than 1% of cases, respectively [9–11].

Other tissues that are rarely affected by hydatid cysts include the perianal area, infratemporal region, or neck [10, 12–14]. It has been shown that the replacement of hydatid cysts in unusual locations might be linked to the parasite genotypes and probably its intragenotypic features [15].

The hydatid cyst of the brain is relatively unusual, and only 2% of CE cases are reported to be in the cerebral location [16].

The development of hydatid cysts in an uncommon location of the body creates a major diagnostic dilemma, and it may mimic other entities that make it challenging to differentiate from other diseases. Above this, the lack of specific radiological findings makes it difficult to differentiate the disease in such cases. Therefore, in any CE-endemic area, a diagnosis of hydatid disease should be suspected in all cystic masses in the body. The current study aimed to find out the rate and features of CE in uncommon locations in Fars Province, Southern Iran, by evaluating the hospital records over a 15-year period.

## 2. Materials and Methods

### 2.1. Patients

The hospital records of patients who underwent surgery for hydatid cysts in university-affiliated hospitals in Fars Province, Southern Iran, for 15 years, from 2004 to 2018, were retrospectively reviewed. Cases were considered as hydatid cyst when the pathological examination confirmed the hydatid cyst by examining the surgically resected specimens postoperatively. Only cases with a definitive diagnosis of hydatid cysts located outside the hepatopulmonary areas, with the unique disease code for hydatid cyst (based on ICD codes: International Statistical Classification of Diseases and Related Health Problems), were included in the data study. For each patient, clinical and demographical data, history of previous hydatid cyst, and cyst features, along with laboratory data and applied treatments were recorded in a data sheet. Hydatid cyst in patients with no history of hepatopulmonary hydatid cyst was considered as primary cases while cases with the previous history of hepatic and/or pulmonary hydatid cyst were considered as secondary cysts.

### 2.2. Ethical Approval

The study was approved by the Ethics Committee of Shiraz University of Medical Sciences (SUMS).

### 2.3. Statistical Analysis

SPSS (SPSS Inc., Chicago, Illinois, USA, version 20) was used for data analysis. Demographics characteristics were analyzed by descriptive data analysis and presented either as mean and standard deviation (SD) or median and interquartile (IQR) as appropriate, based on normality of distribution. Chi-square or Fisher's exact test was utilized to find out the differences among the categorical variables.

## 3. Results

During a 15-year period, between 2004 and 2018, a total of 501 patients were surgically treated for hydatid cysts in two main referral hospitals in Fars Provinces, Southern Iran. Out of these 501 cases, 46 (9.2%) were presented with the unusual location of hydatid disease.

Males constituted 28 (60.9%) of unusual location cases while 18 (39.1%) of the patients were females. The patients' age ranged from 5 to 80 years (mean = 40.49; SD = 20.37), with the highest frequency (20%) among 20 to 29 years and also 50–59 years of age. The patients' hospital admission duration ranged from 3 to 55 days (mean = 11.77, SD = 10.51).

To evaluate the area of the cyst in our study, we decided to calculate the square centimeters (cm^2^) of the area of cysts based on the area of the oval/circle formula; largest radius (half of the largest diameter) multiplied by the smallest radius (half of the smallest diameter) multiplied by Pi (3.14). The size of the cysts ranged from 2 to 20 cm (mean = 8.69, SD = 4.59) for the largest diameter of the cyst, while the area of the cysts ranged from 3 to 314 cm^2^ (median = 32.97, IQR = 18.37–78.5).  Table 1 demonstrates the demographic and clinical features of patients with unusual locations of hydatid cyst in our study.

The most common unusual location for the hydatid cyst was the spleen (30.4% of unusual location cases), followed by the pelvic cavity (15.2%). Out of 46 cases with unusual locations of hydatid cyst, 10 (21.7%) cases had pulmonary, 22 (47.8%) cases had hepatic, and 5 (10.9%) cases had both hepatic and pulmonary hydatid cyst, simultaneously with cysts in unusual locations.  Table 2 shows the unusual locations of hydatid cyst, as well as the simultaneous presence of cysts in unusual and common (liver and lung) locations.

Among the patients, 2 (4.3%) had the following occurrence of a pulmonary hydatid cyst after being treated for a hydatid cyst of an extrapulmonary location (one cardiac and the other spleen). Also, 3 (6.5%) had the following occurrence of hepatic hydatid cyst after treating the cyst in an unusual location (one spleen, one heart, and one subdiaphragmatic area). Also, 3 (6.5%) of the patients (one pelvic cavity, one peritoneal cavity, and one with mesentery of terminal ileum hydatid cyst) had a previous history of hydatid cyst of the liver.

Based on the radiological evaluations for hydatid cyst, out of 19 computed tomography (CT) scans conducted for the evaluation of hydatid cyst in other locations, all were positive and were characteristic and highly suspected of hydatid cyst (sensitivity 100%). However, based on the sonography evaluations, 25 out of 27 (92.6%) were characteristic and highly suspect of hydatid cyst. Also, in two cases (one cardiac and one paravertebral (extradural) hydatid cyst), magnetic resonance imaging (MRI) had characteristic and highly suspected of hydatid cyst.

Regarding the surgical treatment of the cases in our study, 17 (37%) underwent radical surgery while 29 (63%) underwent conservative surgery. Regarding the surgical procedures, 24 (52.2%) underwent laparotomy, 16 (34.8%) evacuation of hydatid cyst, 8 (17.4%) unroofing, 6 (13%) excision, 5 (10.9%) resection, 3 (6.5%) drainage, and 13 (28.3%) underwent organ removal.  Table 3 demonstrates the surgical procedures performed in the current study.

Among the primarily unusual location hydatid cysts, during the mentioned period of our study, only one case of relapse in another unusual location (other than the lung and liver) was observed which developed primarily in the liver and subdiaphragmatic area and relapsed after 3 years in the mesothelium of the terminal ileum.

## 4. Discussion

Hydatidosis is considered a re-emerging disease even in regions that used to have a low prevalence of the disease. This may be due to the lack of preventive measures caused by economic problems and also lack of resources, subsequently increasing the concern for public health issues [17]. Hydatid cyst may affect all organs of the human body but is commonly located in the liver and lungs. This is because the liver is the first sieve that meets the hepatic portal flow where most of the larvae are retained and the cyst starts to grow. Those larvae that cross the liver barrier enter the lungs, where they settle and form hydatid cysts. In a small percentage of cases (10%–20%), the larvae will be able to pass through the liver and lungs and enter the systemic circulation and settle in any tissues other than hepatopulmonary.

In a systematic review by Gramizadeh in 2013, from 463 published cases of the hydatid cyst during 20 years, in the unusual location from Iran, the most common locations were reported to be the CNS, musculoskeletal system, heart, and kidney, whereas some less common locations were the spleen, pancreas, appendix, thyroid, salivary gland, adrenal gland, breast, and ovary [18]. A study of 329 cases of hydatid cysts in Turkey showed that 72 (21.9%) of them were in unusual locations which were mainly in spleen followed by bones, CNS, soft tissue, the kidney, and the gall bladder, respectively [11]. In a study by Cakir et al. in Turkey, a total of 157 patients with unusual locations of hydatid cysts were retrospectively evaluated and the most involved organ was reported to be the spleen [19].

After the liver and lungs, the most common organ involved in hydatid cysts is usually the spleen. Spleen infestation usually occurs by the arterial route if the parasite passes through pulmonary and hepatic filters or via a retrograde venous path in patients with portal hypertension, bypassing the liver and lung [20, 21]. Furthermore, disseminated disease may also result in spleen and peritoneal hydatid cyst as well [22, 23].

In our study, out of 46 hydatid cysts in unusual locations, 30.4% of cases were belonging to the spleen which is 2.8 percent of all reported hydatid cysts (501) in this region. Splenic hydatid cyst may present as a secondary cyst as a result of the rupture of a previous cyst, yet primary are frequently reported [8, 19, 24, 25]. In a case series of unusual sites of hydatid cyst in Iran, the splenic cyst was reported in 20 out of 463 cases (4.3%) of the unusual site for hydatid cysts [18]. In a study by Ahmadi and Hamidi in Hamedan Province, Western Iran, the spleen and kidneys, followed by the peritoneal cavity, were the most unusual affected organs [26].

Spinal hydatid cysts often have unusual clinical presentations sometimes imitating malignant tumors. Such cases have been misguided as a spinal tumor on radiological examination and were diagnosed as hydatid cyst on fine-needle aspiration and histopathological examination [27].

Bone hydatid cyst, which mostly affects the spine, accounts for 0.5% to 2.5% of hydatid cyst cases [10, 11, 28, 29]. The bone hydatid cyst has been reported in 44 patients for 20 years in Iran [18]. In our study, no case of bone hydatid cyst has been detected, and the main reason for that is that the orthopedic patients usually undergo surgeries, including the bone hydatid cyst in a different hospital which has not been included in our study. This should be considered as a limitation of this study.

Cardiac hydatid cyst accounts for about 0.5–2.5% of all hydatid cyst cases in humans [28, 30]. It usually affects the left ventricle. The cardiovascular system was the third most common unusual location of the hydatid cyst reported from Iran [18]. The ventricular wall was reported as the main location for the cardiac hydatid cysts [18]. In the current study, cardiac hydatid cysts were seen in four cases which affected the epicardium, post of the left ventricle, right atrium, and right lateral ventricle.

Soft tissue hydatid disease is rare and occurs in 1% to 4% of hydatid cyst patients. Muscles are not suitable for hydatid cysts to grow, yet hydatid cyst of Psoas muscle, inguinal area, and proximal muscles of the upper and lower limbs are reported in the literature [31–34].

In the current study, a kidney hydatid cyst was seen in 2.1% of cases. In Geramizadeh's study, hydatid cyst of the kidney and the urinary tract was reported in 31 out of 463 (6.7%) cases, reported in the literature [18].

In our series, there were some very rare locations of hydatid cysts including the common bile duct, diaphragm, and pancreas.

In a systematic review by Sokouti et al. [35] comparing laparoscopic versus open surgery in liver hydatid cyst reported no significant difference between the two techniques whereas another study conducted by Polat suggested the use of laparoscopy in the liver and spleen hydatid cyst when cysts are superficial and small in size [36]. However, the laparoscopic approach was not performed in our center, and the management of cysts was mainly focused on laparotomy. Based on the controversial reports in this regard, further studies in this area are justified.

The center in which our study was performed is a referral center in Southern Iran which results in a high number of patients visiting due to various complaints. Since hydatidosis is endemic in our country, this differential diagnosis should not be neglected in patients with complaints addressing other locations than the lung and liver, in which the cyst usually occurs and should especially be suspected in patients with radiological features in favor of cystic structures. Furthermore, in cases of uncommon hydatid cyst, evaluating other locations should be taken into consideration for any infestations of hydatidosis.

## 5. Conclusions

In CE-endemic areas, hydatid disease can affect any organ, from head to toe, in humans. The disease should be considered in the differential diagnosis of any cystic entities anywhere in the body. Spleen and pelvic cavity should be considered as the most unusual locations of hydatid cyst in any CE-endemic area. Besides, hospital records lack information about hydatid cyst imaging details, which must be carefully noted in each patient's record.

## Figures and Tables

**Table 1:** Demographic and clinical features of patients diagnosed with hydatid cyst of unusual locations.

Variable	Frequency	Percent
Age groups (years)		
<10	2	4.4
10–19	6	13.3
20–29	9	20
30–39	5	11.1
40–49	5	11.1
50–59	18	40
60–69	6	13.3
≥70	3	6.7

Gender		
Male	28	60.9
Female	18	39.1

Hospitalization duration (days)		
≤5	16	36.4
6–10	11	25
11–15	6	13.6
16–20	2	4.5
21–30	7	15.9
>30	2	4.5

Number of cysts		
1	21	45.7
2	7	15.2
≥3	4	8.7

Size of cyst, largest diameter (cm)		
≤5	7	21.2
6–10	18	54.5
11–15	4	12.1
≥16	4	12.1

Area of the cyst (cm^2^)		
<26	14	43.8
26–50	5	15.6
51–75	2	6.3
76–100	7	21.9
>100	4	12.5

**Table 2:** Frequency of unusual locations of hydatid cyst and the simultaneous presence of cysts in unusual and common (liver and lung) locations.

Location	Frequency (%)	Simultaneous common hydatid cyst (% of total)	
		Lung	Liver
Spleen	14 (30.4)	2 (4.3)	6 (13)
Pelvic cavity	7 (15.2)	1 (2.2)	6 (13)
Heart	4 (8.7)	2 (4.3)	0 (0)
Epicardium	1 (2.2)	1 (2.2)	0 (0)
Post of left ventricle	1 (2.2)	1 (2.2)	0 (0)
Right atrium	1 (2.2)	0 (0)	0 (0)
Right lateral ventricle	1 (2.2)	0 (0)	0 (0)
Subdiaphragmatic area	5 (10.9)	2 (4.3)	3 (6.5)
Abdominal and peritoneal cavity	5 (10.9)	0 (0)	3 (6.5)
Abdominal cavity	1 (2.2)	0 (0)	1 (2.2)
Abdominal wall	1 (2.2)	0 (0)	1 (2.2)
Peritoneal cavity	1 (2.2)	0 (0)	1 (2.2)
Peritoneum	1 (2.2)	0 (0)	0 (0)
Retroperitoneal cavity	1 (2.2)	0 (0)	0 (0)
Common bile duct	2 (4.3)	1 (2.2)	0 (0)
Kidney	1 (2.2)	0 (0)	1 (2.2)
Mediastinum	2 (4.3)	1 (2.2)	1 (2.2)
The mesentry of the terminal ileum	1 (2.2)	0 (0)	1 (2.2)
Ovary	1 (2.2)	0 (0)	0 (0)
Pancreas	2 (4.3)	1 (2.2)	1 (2.2)
Cerebral	1 (2.2)	0 (0)	0 (0)
Paravertebral (extradural)	1 (2.2)	0 (0)	0 (0)
Psoas muscle	1 (2.2)	0 (0)	1 (2.2)
Inguinal area	1 (2.2)	0 (0)	0 (0)

**Table 3:** Surgical procedures performed regarding hydatid cysts in uncommon locations.

Location	Surgical procedure (% within the organ)						
	Evacuation	Excision	Resection	Laparotomy	Drainage	Unroofing	Removal of organ
Spleen	5 (35.7)	1 (7.1)	0 (0)	7 (50)	1 (7.1)	2 (14.3)	5 (35.7)
Pelvic cavity	4 (57.1)	3 (42.9)	1 (14.3)	2 (28.6)	0 (0)	2 (28.6)	0 (0)
Heart	0 (0)	1 (25)	1 (25)	0 (0)	0 (0)	0 (0)	0 (0)
Subdiaphragmatic area	2 (40)	0 (0)	0 (0)	4 (80)	2 (40)	1 (20)	0
Abdominal and peritoneal cavity	2 (40)	2 (40)	2 (40)	5 (100)	0 (0)	0 (0)	2 (40)
Common bile duct	0 (0)	0 (0)	0 (0)	1 (50)	2 (100)	0 (0)	0 (0)
Kidney	1 (100)	0 (0)	0 (0)	1 (100)	1 (100)	1 (100)	0 (0)
Mediastinum	1 (50)	0 (0)	0 (0)	0 (0)	2 (100)	0 (0)	0 (0)
The mesentry of the terminal ileum	0 (0)	0 (0)	0 (0)	0 (0)	1 (100)	0 (0)	0 (0)
Ovary	0 (0)	0 (0)	0 (0)	1 (100)	1 (100)	0	0 (0)
Pancreas	0 (0)	0 (0)	0 (0)	2 (100)	2 (100)	1 (50)	1 (50)
Paravertebral (extradural)	1 (100)	0 (0)	0 (0)	0 (0)	1 (100)	0 (0)	0 (0)
Psoas muscle	0 (0)	0 (0)	1 (100)	1 (100)	1 (100)	1 (100)	1 (100)
Inguinal area	0 (0)	0 (0)	0 (0)	1 (100)	1 (100)	0 (0)	0 (0)

## Data Availability

The nominal and ordinal data used to support the findings of this study are available from the corresponding author upon request.
